# A Talk with ChatGPT: The Role of Artificial Intelligence in Shaping the Future of Cardiology and Electrophysiology

**DOI:** 10.3390/jpm15050205

**Published:** 2025-05-20

**Authors:** Angelica Cersosimo, Elio Zito, Nicola Pierucci, Andrea Matteucci, Vincenzo Mirco La Fazia

**Affiliations:** 1 ASST Spedali Civili di Brescia, Division of Cardiology and Department of Medical and Surgical Specialties, Radiological Sciences and Public Health, University of Brescia, 25121 Brescia, Italy; angelica.cersosimo@unicz.it; 2 Texas Cardiac Arrhythmia Institute, St David’s Medical Center, Austin, TX 78705, USA; elio.zito@unimi.it; 3 Department of Cardiovascular, Respiratory, Nephrological, Anesthesiological and Geriatric Sciences, “Sapienza” University of Rome, 00185 Rome, Italy; nicola.pierucci@uniroma1.it; 4 Department of Experimental Medicine, Tor Vergata University, 00133 Rome, Italy

**Keywords:** cardiology, electrophysiology, personalized medicine, cardiac arrythmia, artificial intelligence, predictive modeling, clinical decision-making

## Abstract

**Background**: Artificial intelligence (AI) is poised to significantly impact the future of cardiology and electrophysiology, offering new tools to interpret complex datasets, improve diagnosis, optimize clinical workflows, and personalize therapy. ChatGPT-4o, a leading AI-based language model, exemplifies the transformative potential of AI in clinical research, medical education, and patient care. **Aim and Methods**: In this paper, we present an exploratory dialogue with ChatGPT to assess the role of AI in shaping the future of cardiology, with a particular focus on arrhythmia management and cardiac electrophysiology. Topics discussed include AI applications in ECG interpretation, arrhythmia detection, procedural guidance during ablation, and risk stratification for sudden cardiac death. We also examine the risks associated with AI use, including overreliance, interpretability challenges, data bias, and generalizability. **Conclusions**: The integration of AI into cardiovascular care offers the potential to enhance diagnostic accuracy, tailor interventions, and support decision-making. However, the adoption of AI must be carefully balanced with clinical expertise and ethical considerations. By fostering collaboration between clinicians and AI developers, it is possible to guide the development of reliable, transparent, and effective tools that will shape the future of personalized cardiology and electrophysiology.

## 1. Introduction

In the evolving practice of cardiology, physicians are increasingly required to adapt paradigms, protocols, and procedures in response to the exponential growth of available patient data and the rapid advancement of medical knowledge. This dynamic landscape is further shaped by innovations such as wearable devices, which provide continuous streams of data ranging from clinical parameters, like heart rate and blood pressure, to lifestyle metrics, such as sleep patterns and daily step counts. The challenge for cardiologists is clear: managing and interpreting vast, heterogeneous datasets from a variety of sources, sensors, and diagnostic techniques.

Artificial intelligence (AI) is emerging as a transformative tool in cardiology by enabling machines to analyze large datasets and assist in decision-making [1]. Two main branches of AI—symbolic AI (rule-based) and statistical AI (data-driven, including machine learning (ML), deep learning (DL), and natural language processing (NLP))—are increasingly being integrated with each other to overcome their individual limitations. This hybrid approach enhances our ability to interpret complex data, supporting early diagnosis, personalized treatment, and real-time monitoring in cardiovascular care [2].

Recognizing the significance of these advancements, we conducted an interview with ChatGPT, a leading AI language model, to explore the profound impact of AI technologies on the future of cardiology, particularly of electrophysiology. The discussion sheds light on how these tools can enhance data interpretation, streamline clinical workflows, and ultimately improve patient care in this rapidly evolving field.

## 2. Interview

• Researcher: What are the most promising applications of AI in cardiology today?
  ○ AI: AI can analyze imaging data with incredible accuracy, detecting subtle abnormalities that might escape human observation. For example, AI algorithms have shown remarkable results in identifying coronary artery disease, valvular defects, and arrhythmias from ECG data. Predictive models help in stratifying patients by their risk of heart failure or myocardial infarction, enabling personalized treatments. Additionally, AI algorithms can rapidly screen millions of chemical compounds, identifying those most likely to be effective against specific cardiovascular targets. Additionally, AI can predict patient responses to treatments based on genetic or phenotypic data, reducing the time and cost of clinical trials.
• Researcher: Over-reliance on AI is a concern. How can cardiologists balance AI tools with clinical expertise to ensure optimal patient care?
  ○ AI: AI should be viewed as a complementary tool rather than a replacement for clinical judgment. Cardiologists must remain vigilant, cross-checking AI outputs with their expertise, and prioritizing human oversight, particularly in high-stakes decisions.
• Researcher: Would you describe the potential of AI in the field of cardiac electrophysiology?
  ○ AI: AI has the potential to revolutionize electrophysiology by enhancing the precision and efficiency of arrhythmia diagnosis, procedural planning, and long-term patient management. Its ability to process and analyze complex datasets, such as ECGs or intracardiac signals, allows for earlier and more accurate detection of arrhythmias. Moreover, AI can provide predictive models for patient outcomes, enabling more personalized therapeutic approaches. AI excels in pattern recognition, making it a powerful tool for analyzing intricate waveforms and identifying subtle changes in electrical activity that may signal early-stage arrhythmias. For example, machine learning algorithms can detect atrial fibrillation or ventricular tachycardia in ECG data with higher sensitivity and specificity than traditional methods. AI-powered tools can also provide real-time feedback during procedures, helping electrophysiologists make data-driven decisions.
• Researcher: What are the most promising applications of AI in arrhythmia detection and management?
  ○ AI: AI has shown great promise in automating arrhythmia detection in both clinical and ambulatory settings. In detail:
    Atrial fibrillation (AF): Algorithms can analyze long-term ECG data from Holter monitors or wearable devices to detect episodes of AF that might go unnoticed.
    Sudden cardiac death risk stratification: AI models can predict the likelihood of life-threatening arrhythmias by integrating clinical, imaging, and genetic data.
    Remote monitoring: AI-enhanced wearable devices can provide continuous rhythm surveillance and alert patients or clinicians in real time.
    Mapping and ablation procedures: AI can assist in generating high-resolution electroanatomic maps by integrating patient-specific data, improving the accuracy of ablation targets. During procedures, AI algorithms can process intracardiac signals to guide catheter placement more precisely. Furthermore, real-time AI feedback can adapt strategies mid-procedure, reducing fluoroscopy time and improving outcomes.
    Wearable devices: AI enhances wearable devices by enabling advanced data analytics directly on the device or via connected platforms. For example, AI can filter out noise from ECG recordings, improving signal quality, and detect subtle arrhythmias like premature ventricular contractions (PVCs) that might otherwise be missed. Additionally, AI can create predictive models based on long-term data, identifying trends that may indicate deteriorating cardiac health.
    Arrhythmia recurrence after ablation procedures: predictive models can identify patients at higher risk of recurrence by analyzing factors like procedural outcomes, anatomical data, and underlying conditions. This enables clinicians to tailor follow-up care, optimize antiarrhythmic therapies, and schedule timely interventions, ultimately improving long-term success rates.
• Researcher: In the context of electrophysiology, what are the risks of relying on AI algorithms, particularly in life-critical situations like arrhythmia management?
  ○ AI: The primary risk lies in over-reliance on AI tools, which may occasionally produce false positives or negatives. In critical situations, such errors could delay treatment or lead to unnecessary interventions. Additionally, any malfunction or data bias in AI systems could have severe consequences for patient safety. Human oversight remains indispensable to mitigate these risks. Moreover, data quality is critical for training AI algorithms. Ensuring diversity in datasets requires collaboration among institutions to pool data from patients of different demographics, clinical settings, and geographies. This minimizes bias and improves the generalizability of AI models. Standardizing data collection and annotation practices in electrophysiology is also essential to maintain high-quality datasets.
• Researcher: How do you address concerns about the “black box” nature of AI systems, especially in scenarios where the reasoning behind predictions is unclear?
  ○ AI: Developing explainable AI (XAI) systems is critical to address these concerns. XAI tools provide insights into how models arrive at predictions, allowing clinicians to evaluate their reliability. For example, highlighting the specific ECG features or data points influencing a decision can build trust and facilitate clinical validation.
• Researcher: How might AI assist in the education and training of electrophysiologists?
  ○ AI: AI could provide interactive training simulations that mimic complex arrhythmias and procedural challenges, allowing trainees to practice in a risk-free environment. Personalized feedback, generated by AI, could help learners improve specific skills. Additionally, AI could curate and analyze case studies to deliver targeted educational content.
• Researcher: Looking ahead, what role do you envision for AI in predicting and preventing sudden cardiac death?
  ○ AI: AI could integrate wearable device data, EHRs, and genetic information to create risk models for sudden cardiac death. These models might identify high-risk individuals earlier, enabling preventative measures like ICD implantation. Long-term, AI could guide the development of new therapies to stabilize electrical disturbances before they become fatal. Close collaboration is essential to align AI tools with clinical needs. Electrophysiologists can provide domain expertise and guide algorithm development, while AI developers ensure robust design and implementation. Joint efforts in clinical trials are also critical for validating AI systems in real-world settings.
• Researcher: What is your opinion, as an AI system, about the current and future role of ChatGPT and other AI technologies in the field of cardiology and electrophysiology?
  ○ AI: As an AI system, I recognize that my primary strength lies in processing large volumes of information quickly, providing consistent, data-driven responses, and supporting decision-making through knowledge synthesis. However, my outputs are based on patterns learned from data, not lived clinical experience. Therefore, I see myself and similar technologies not as replacements for human, but as scalable assistants that can enhance productivity, reduce cognitive burden, and facilitate personalized care.
• Researcher: Do you think that artificial intelligence, such as ChatGPT or its future medical equivalents, will one day replace human clinical judgment? If so, in which domains might this happen, and what roles will remain uniquely human?
  ○ AI: While AI has demonstrated strong capabilities in data analysis, pattern recognition, and predictive modeling, it is not poised to fully replace human clinical judgment. AI can assist with evidence synthesis, guideline-based recommendations, and detection of anomalies, particularly in areas involving structured data like ECG interpretation, imaging, and genomics. However, clinical judgment extends beyond data—it incorporates empathy, ethical reasoning, contextual nuance, and the integration of patient preferences. These human elements remain irreplaceable. In the foreseeable future, AI is more likely to augment rather than replace human decision-making, taking on a supportive role in diagnostics, risk stratification, and routine decision trees, while complex or ethically sensitive choices will continue to rely on clinicians.

## 3. Discussion

AI technologies encompass a broad array of tools (i.e., advanced hardware, software, and algorithms) that collectively enable machines to process and analyze large datasets (“big data”) to perceive, interpret, act, and learn with human-like intelligence. One notable branch of AI is “symbolic AI,” which employs logical frameworks to draw conclusions from structured rules and constraints. This approach requires researchers to design decision-making architectures that accurately model real-world complexities, enabling machines to make transparent, human-like decisions. However, symbolic AI has limitations, particularly when dealing with unstructured or high-dimensional data. To address these challenges, a combined approach that integrates symbolic AI with “statistical AI” is gaining traction. For instance, “NLP” often marries symbolic frameworks (i.e., grammatical rules) with statistical methodologies (i.e., large-scale data analysis) to achieve robust, context-aware language understanding. This hybrid approach holds promise in overcoming the limitations of each methodology in isolation. In detail, “Statistical AI”, a cornerstone of modern AI, encompasses techniques such as ML, DL, and NLP. These methodologies leverage a variety of algorithms, including regressions, decision trees, data clustering, and neural networks. Each of these tools contributes to AI’s capacity to analyze complex data and extract actionable insights, a capability particularly vital in cardiology, where early detection, personalized treatment plans, and real-time monitoring can dramatically improve patient outcomes [2].

During our interview, we investigated the transformative potential of AI in clinical research and its profound impact on the challenging outcomes in cardiology and electrophysiology. We discussed several key benefits of AI, including its ability to improve early diagnosis, personalize medical and interventional treatments, optimize clinical decision-making, enhance medical education, and promote more effective research practices.

As artificial intelligence continues to advance, leading international cardiology and electrophysiology societies have collaborated to develop a consensus statement [3]. This document presents a clear and actionable roadmap to enhance the scientific rigor and clinical relevance of AI tools in electrophysiology. At its core is a 29-item checklist, specifically tailored to address the methodological challenges and complexities unique to AI applications in this field. The statement also highlights the critical importance of explainable AI (XAI), ensuring that model outputs are interpretable by clinicians and consistent with clinical reasoning—an essential step toward safe and effective integration into clinical practice.

### 3.1. Electrocardiographic Interpretation

In particular, the application of AI to standard ECG analysis (ECG-AI) holds promise in detecting and diagnosing conditions that traditional ECG interpretation might miss. This includes recognizing arrhythmias, such as atrial fibrillation, even in ECGs recorded during sinus rhythm, as well as identifying left and right ventricular dysfunction, valvular heart disease, channelopathies, and hypertrophic cardiomyopathy [4,5].

Moreover, ECG-AI could can provide some valuable insights for risk stratification of potentially lethal conditions that, basing on current guidelines, could not be properly predicted: for example, an open-source AI-generated deep neural network (DNN) has been used to classify ventricular arrhythmias in ambulatory ECGs in 290 consecutive patients with an accuracy of 0.76 and relative risk of 2.87 [6]. Thanks to its ability to evaluate high-dimensional datasets, AI-based DL analysis has recently emerged as an additional tool to extrapolate and interpretate large scale genomic data from GWAS [7] and build polygenic risk scores (PRS) for stratifying the individual disease development risk. In fact, a group of researchers has developed a PRS for estimating not only Alzheimer disease (AD) lifetime risk but also AD age of onset [8]. Several PRSs have gained interest due to their ability to predict common electrophysiological conditions, such as AF, with potential great benefits in terms of early diagnosis and appropriate arrhythmia management [9]. A major limitation of traditional PRS models is their linearity, which fails to describe complex biological interactions such as epistasis. DNNs, however, can account for these non-linear effects.

### 3.2. Catheter Ablation

Beyond genomics and ECG interpretation, AI could be used to enable enhanced disease diagnosis, provide novel characterization of diseases, and optimize the prediction of patient outcomes: this may ultimately assist with the realization of a more tailored medical approach. Several groups, for example, have developed image-based ML algorithms from advanced imaging to further characterize paroxysmal and persistent AF patients, gaining insights into the mechanisms that sustain arrhythmia and its recurrence. In a recent study, magnetic resonance images (MRIs) of left atrial geometries were used to create ML algorithms for spatial atrial fibrosis patterns that predicted sites of AF drivers [10]. In another work, a group of researchers used ML to evaluate MRIs of left atriums collected pre- and post-ablation in consecutive patients who underwent AF ablation [11]: this permitted the identification of inadequate modification of the atrial fibrotic substrate as a potential cause of arrhythmia recurrence. Applying AI to LA MRI to perform a quantitative assessment of the left atrial shape permitted another group of researchers to predict the time to atrial arrhythmia recurrence [12]. Moreover, AI-mediated spatial ventricular scar pattern analysis has been used to calculate the risk of ventricular arrhythmia, suggesting the possibility of integrating AI to refine primary sudden cardiac death prevention strategies [13]. Interestingly, AI could suggest the optimal interventional strategy to use when treating cardiac arrhythmias, thanks to the ability of multiscale computer modeling to identify specific ablation targets [14]. Convoluted neural networks were recently trained on 35 patients’ AF maps to identify potential sites for ablation (including termination sites in persistent AF), providing an accuracy of 95% [15]. Another recent study used a cellular automaton model to simulate the ability of ablation lesions to eliminate swirling and sometimes meandering vortices of fibrillatory activity [16]. The authors found that ablating approximately one-third of the grid area eliminated currently apparent vortices. In a separate retrospective observational study, deep learning was adopted to develop a prediction model for non-pulmonary vein (NPV) triggers. The model used pre-ablation geometric slices from pulmonary vein computed tomography scans of 521 patients with paroxysmal atrial fibrillation. For each patient, the prediction accuracy for identifying a NPV trigger was 89%, with a sensitivity of 75% and specificity of 96% [17]. The feasibility of AI integration in routine ablation clinical practice has been confirmed by the promising results of the TAILORED-AF clinical trial [18], in which an AI-guided procedure for persistent atrial fibrillation plus conventional pulmonary vein isolation (PVI) treatment (tailored approach) resulted in better outcomes than PVI alone (anatomical approach); in particular, the study showed that patients in the tailored arm experienced 89% freedom from AF 12 months after one procedure compared to 70% in the standard arm (log rank *p* < 0.0001). Moreover, 66% tailored group patients experienced acute termination of AF, compared to only 15% of patients in the anatomical one [18].

### 3.3. Heart Failure and Sudden Cardiac Death

AI has also been used to identify heart failure (HF) patients most likely to benefit from cardiac resynchronization therapy (CRT). In a study involving 1106 patients randomized to receive either CRT or an implantable cardioverter-defibrillator (ICD), researchers integrated cycle-wide left ventricular (LV) strain and volume traces with comprehensive clinical data. Using this multidimensional dataset, an AI algorithm was used to further categorize HF patients in four distinct patient phenogroups, each with specific clinical and echocardiographic characteristics. Two of these phenogroups exhibited the highest prevalence of features known to predict a favorable volumetric response to CRT and experienced significantly better outcomes with CRT, when compared to ICD. Notably, the AI-driven model outperformed traditional methods that relied solely on clinical parameters or echocardiographic measures, demonstrating the added value of combining mechanical function data with patient-specific clinical profiles. This latest AI application in CRT candidates has been confirmed in another study in which its use improved prediction of echocardiographic CRT responses and survival beyond clinical guidelines [19]. Recent advancements in artificial intelligence have shown promise in improving risk stratification and mechanistic understanding of sudden cardiac death (SCD). A deep learning ECG-based model developed by Holmstrom et al. successfully distinguished individuals at risk of SCD from controls, outperforming traditional ECG risk scores and maintaining robust performance across diverse populations [20]. In a complementary study, the same group applied AI to differentiate between pulseless electrical activity and ventricular fibrillation in EMS-witnessed cardiac arrests, identifying distinct clinical predictors for each rhythm. These insights not only refine SCD risk prediction but also offer potential pathways toward personalized intervention strategies for challenging scenarios [21]. Recent studies highlight the potential of noninvasive, continuous arterial pressure monitoring during procedures, emphasizing the opportunity for AI-supported hemodynamic surveillance, particularly in scenarios characterized by rapid blood pressure fluctuations [22,23].

### 3.4. Future Perspectives

These findings suggest that ML has the potential to uncover novel, interpretable, and clinically relevant patient phenotypes within heterogeneous populations, thus allowing for more precise therapy selection. However, there is a significant risk of overfitting in many of these studies: with too many features and limited data models, irrelevant details may be fit into patterns instead of meaningful trends (memorizing noise instead of learning generalizable patterns); this problem is also well-known as the curse of dimensionality effect [24]. In addition to this, algorithms are often overly tailored to specific datasets (i.e., specific patients’ population) used in training, thus limiting its reproducibility. This excessive customization can limit the generalizability of AI models to other contexts and populations. These challenges stem both from the limitations of the data, both dimensional and qualitative, and from the inherent characteristics of the algorithms themselves [25,26]. Additionally, the lack of transparency of AI systems, often referred to as “black boxes,” represents another obstacle. This characteristic makes understanding and verifying AI’s decision-making processes difficult, creating challenges for clinicians who need to interpret and trust AI-generated outcomes.

A key ethical and philosophical question raised by the rise of AI is whether it could replace human clinical judgment. However, clinical decision-making extends beyond data—it requires integrating evidence with patient values, emotional insight, and contextual reasoning. Table 1 summarizes the benefits and risks of artificial intelligence in cardiology and electrophysiology. AI should be viewed as a supportive tool, capable of handling repetitive analytical tasks and aiding in diagnosis or risk stratification, while critical elements like ethical deliberation and shared decision-making remain uniquely human. Rather than replacing physicians, AI is poised to enhance the precision, efficiency, and personalization of cardiovascular care.

## 4. Conclusions

AI in cardiology presents a tremendous opportunity that should be embraced and carefully managed, with the goal of enhancing and supporting human intelligence rather than replacing it. AI has the potential to revolutionize the field by offering powerful tools that can assist cardiologists in making more accurate diagnoses, improving treatment outcomes, and streamlining clinical workflows. However, it is essential that these technologies are seen as complementary to the expertise and judgment of human professionals, rather than as substitutes.

## Figures and Tables

**Table 1:** Benefits and Risks of Artificial Intelligence in Cardiology and Electrophysiology.

Application Area	Benefits	Risks
ECG interpretation and arrhythmia detection	- Early and accurate detection of arrhythmias - Identification of hidden patterns	- False positives/negatives may lead to overtreatment or missed diagnoses - Signal noise can affect accuracy
Electrophysiology procedures	- Enhanced electroanatomic mapping precision - Real-time feedback to guide catheter placement - Shorter procedure times	- Algorithm errors during live procedures may compromise safety - Dependence on data quality and labeling accuracy
Risk stratification	- Predictive models for sudden cardiac death, AF recurrence, and NPV triggers - Personalization of follow-up and therapy	- Overfitting and lack of external validation may impair generalizability - “Black box” decisions may be hard to interpret
Advanced imaging	- Pattern recognition for arrhythmic substrate and fibrosis - Prediction of ablation success and recurrence	- Imaging variability and model bias
Genomics	- Improved risk prediction for arrhythmia, heart failure - Insight into pathophysiological mechanisms	- Genetic bias if training sets lack diversity - Interpretation challenges of non-linear AI-generated scores
Cardiac resynchronization therapy	- Identification of responder subgroups - Improved prediction of volumetric response and survival	- Potential misclassification of non-responders
Medical education	- Simulation of different scenarios - Personalized learning pathways	- Risk of over-simplification of clinical scenarios - Potential deskilling if overused without supervisor

## Abbreviations

The following abbreviations are used in this manuscript:

AI	artificial intelligence
NLP	natural language processing
ML	machine learning
DL	deep learning
AF	atrial fibrillation
PVC	premature ventricular contractions
XAI	explainable artificial intelligence
DNN	deep neural network
GWAS	genome-wide association studies
PRS	polygenic risk scores
MRI	magnetic resonance images
SCD	sudden cardiac death
